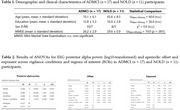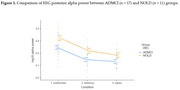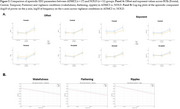# Periodic and Aperiodic EEG Parameters During Transitions from Wakefulness to Light Sleep: Preliminary Results on Patients with Mild Cognitive Impairment Due to Alzheimer's Disease vs. Healthy Elderly

**DOI:** 10.1002/alz70856_106405

**Published:** 2026-01-11

**Authors:** Matteo Carpi, Enrico Michele Salamone, Claudio Del Percio, Roberta Lizio, Susanna Lopez, Giuseppe Noce, Mina De Bartolo, Veronica Henao Isaza, Antonio Pio Afragola, Burcu Bölükbaş, Lorenc Barjami, Bahar Güntekin, Görsev Yener, Claudio Babiloni

**Affiliations:** ^1^ Sapienza University of Rome, Rome, Rome, Italy; ^2^ Oasi Research Institute – IRCCS, Troina, Italy, Troina, Italy, Italy; ^3^ Sapienza University of Rome, Rome, Italy; ^4^ IRCCS Synlab SDN, Naples, Italy; ^5^ Sapienza University of Rome, Roma, Italy, Italy; ^6^ Grupo Neuropsicología y Conducta, Facultad de Medicina, Universidad de Antioquia, Medellín, Colombia; ^7^ Sapienza University of Rome, Roma, Rome, Italy; ^8^ Istanbul Medipol University, Istanbul, Turkey; ^9^ Dokuz Eylül University, Balçova, Izmir, Turkey; ^10^ San Raffaele Cassino, Cassino, Italy

## Abstract

**Background:**

The periodic (e.g., EEG alpha power density) and aperiodic (offset and slope of EEG power density) components of resting‐state EEG rhythms reflect different aspects of global neural dynamics and have been linked to excitatory/inhibitory balance. This study investigates these components across vigilance stages (wakefulness, flattening, ripples) in patients with mild cognitive impairment due to Alzheimer's disease (ADMCI) compared to healthy elderly (NOLD).

**Method:**

Spectral analysis was performed on EEG data recorded from 19 scalp electrodes during a ∼30‐minute session in age‐, sex‐, and education‐matched ADMCI and NOLD participants (*n* = 17 vs. *n* = 11) showing transitions from quiet wakefulness (wakefulness stage, characterized by dominant posterior alpha activity at 8–12 Hz) to light sleep (flattening stage, marked by reduced EEG amplitude, and ripples stage, with diffuse theta activity at 4–7 Hz) based on a modified version of Hori's sleep onset classification. Periodic (spectral power in the 8–12 Hz alpha band for posterior electrodes) and aperiodic (offset and slope in the 3–40 Hz range) parameters were analyzed across vigilance stages. ANOVA was performed with Group, Stage, and Region of Interest (Frontal, Central, Temporal, Posterior) as factors.

**Results:**

For the periodic EEG component, posterior alpha power density showed a significant Group effect, indicating reduced posterior alpha activity from quiet wakefulness to light sleep in ADMCI compared to NOLD (*p* < 0.01). In contrast, offset and exponent exhibited significant Condition effects, reflecting increased cortical inhibition (higher offset and exponent) across vigilance stages (*p* < 0.001) with no significant Group differences. Region of Interest effects showed greater inhibition across stages in parietal‐occipital regions compared to anterior regions (*p* < 0.001).

**Conclusion:**

In this study, periodic EEG alpha power was the most sensitive marker of vigilance dysfunctions in ADMCI, while aperiodic EEG parameters primarily captured a general increase in inhibition during transitions from wakefulness to light sleep, independent of disease. Vigilance‐related changes in cortical inhibition do not appear to be a hallmark of prodromal Alzheimer's disease, which is instead characterized by altered oscillatory, frequency‐specific activity. Future research should integrate both periodic and aperiodic EEG features to enhance the understanding of neurophysiological dynamics in Alzheimer's disease.